# The molecular mechanisms of IUGR programmed adulthood cardiovascular disease

**DOI:** 10.3389/fcell.2025.1589038

**Published:** 2025-05-15

**Authors:** Ting Wu, Wen Zhang, Yangong Wang, Hong Luo, Yifei Li

**Affiliations:** ^1^ Key Laboratory of Birth Defects and Related Diseases of Women and Children of MOE, West China Second University Hospital, Sichuan University, Chengdu, Sichuan, China; ^2^ Department of Ultrasonic Medicine, West China Second University Hospital, Sichuan University, Chengdu, Sichuan, China; ^3^ Minhang Hospital, Institutes of Biomedical Sciences, Fudan University, Shanghai, China; ^4^ Department of Pediatrics, West China Second University Hospital, Sichuan University, Chengdu, Sichuan, China

**Keywords:** IUGR, cardiovascular disease, programmed diseases, molecular mechanisms, epigenetic modification, therapy

## Abstract

Intrauterine growth restriction (IUGR) is secondary to several maternal and fetal adverse conditions. Recently, there is a convincing association between the onset of IUGR and adulthood programmed complications. Among them, the disorders in the cardiovascular system have been revealed by a series of researches. Currently, the prevalence of IUGR is considered to be related to programmed hypertension, coronary artery lesions, pulmonary hypertension, metabolic dysfunction, and even heart failure. According to the emerging knowledge in this field, the experiences of IUGR would induce prolonged inflammation, oxidative injuries, aberrant metabolites and epigenetic regulation, which resulted in endothelial, smooth muscle cells and cardiomyocytes damages. In this review, we summarized the evidences and progress in establishing the association between IUGR and programmed cardiovascular diseases and involved molecular mechanisms. Furthermore, we also discussed the potential efficient therapeutic strategies. This comprehensive review demonstrated that IUGR manifested long-term consequences persisting into adulthood through multifaceted molecular pathways, notably oxidative stress mechanisms, mitochondrial dysfunction, and epigenetic alterations. These findings underscored the critical importance of implementing early preventive interventions and developing personalized therapeutic approaches in future clinical practice.

## Introduction

**FIGURE 1:**
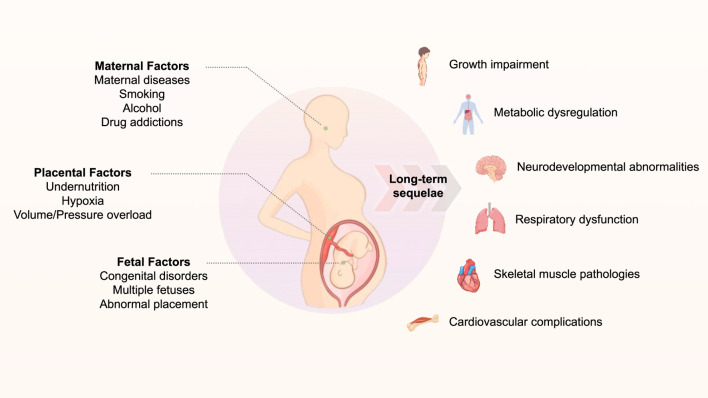
The schematic diagraph of the association between gestational adverse events and long-term related diseases.

Intrauterine growth restriction (IUGR) is characterized by fetal growth that fails to achieve its genetic potential due to multiple etiological factors, encompassing placental, maternal, and fetal components, including placental insufficiency, maternal pathological conditions, and fetal genetic anomalies. This condition affects approximately 10% of pregnancies worldwide, with a notably higher prevalence of approximately 27% of all live births in developing nations, resulting in both immediate and long-term implications for offspring health (Lopez-Tello et al., 2019; Black, 2015) In the acute phase, IUGR significantly elevates morbidity and mortality rates during fetal, perinatal, and neonatal periods. Chronically, owing to exposure to adverse intrauterine conditions and subsequent postnatal catch-up growth, IUGR can precipitate numerous long-term sequelae, including growth impairment, metabolic dysregulation, neurodevelopmental abnormalities, respiratory dysfunction, skeletal muscle pathologies, and cardiovascular complications (Kesavan and Devaskar, 2019; Li P. et al., 2022; Oliveira et al., 2021) (Figure 1). This review specifically addresses cardiovascular disease manifestations in adults with a history of IUGR. Contemporary evidence strongly supports that the intrauterine environment serves as a critical foundation for cardiovascular development, thereby explaining the enhanced susceptibility to cardiovascular disease in IUGR-affected adults (Rock et al., 2021). Furthermore, retrospective epidemiological investigations have demonstrated that IUGR increases the propensity for metabolic disorders in offspring, including diabetes, obesity, dyslipidemia, and insulin resistance, which collectively contribute to the heightened prevalence of cardiovascular diseases in adulthood (Barker, 2006; McMillen and Robinson, 2005).

## Fetal programmed adulthood diseases

An individual’s health trajectory is shaped by cumulative life experiences, with events occurring during early developmental periods exerting particularly profound influences on long-term health outcomes (Cheong et al., 2016). This concept is encapsulated in the fetal programming hypothesis of adult disease, which postulates that gestational stress events induce alterations in cardiovascular homeostatic regulation, thereby predisposing offspring to cardiovascular disease (Scherrer et al., 2015). IUGR, defined as the failure to achieve genetically predetermined growth potential, is widely recognized as a primary prenatal risk factor for adult cardiovascular disease. However, the underlying mechanistic pathways linking fetal programming to adult cardiovascular disease in IUGR, and their associations with offspring health trajectories, remain incompletely elucidated. Therefore, this review examines the spectrum and risk factors of adult cardiovascular disease in IUGR, synthesizes potential mechanisms, and explores prospective therapeutic interventions to ameliorate cardiac outcomes.

Fetal programming represents an adaptive process that establishes crucial links between adverse intrauterine environments and chronic pathological conditions in adulthood (Barker, 2006; Pike et al., 2008). This paradigm was initially proposed by Dr. David Barker in 1990, highlighting the critical importance of early-life programming in determining long-term health outcomes (Barker, 1990). While fetal programming initially serves as a beneficial adaptive response to maintain fetal growth during gestation, epidemiological evidence suggests that genetic and environmental factors, including nutritional imbalances and maternal metabolic perturbations, can trigger programming mechanisms that precipitate adverse long-term health consequences in offspring. These outcomes encompass cardiovascular disease, metabolic syndrome, neurological disorders, and reproductive and immune dysfunction (Reynolds et al., 2019; Alexander et al., 2015; Drever et al., 2010; Rasmussen et al., 2022). Marciniak’s review emphasized the potentially pivotal role of epigenetic modifications in fetal programming, whereby adverse intrauterine environmental factors can induce alterations in fetal gene expression patterns, ultimately contributing to increased chronic disease susceptibility in later life (Marciniak et al., 2017). Given that cardiovascular disease remains a leading cause of adult mortality, this area warrants particular attention and investigation.

## The relationship between Iugr and programmed adulthood CVD

Although multiple factors contribute to the pathogenesis of adult cardiovascular diseases, exposure to adverse events during early life, particularly in the prenatal period, has been demonstrated to significantly correlate with disease onset in later life. Adult-onset cardiovascular conditions, including stroke, hypertension, heart failure and coronary artery disease, as well as circulatory system-related mortality, demonstrate strong associations with IUGR and low birth weight cohorts. Compelling epidemiological evidence supports this correlation. In a seminal study, Martyn and colleagues investigated a cohort of 50-year-old individuals in Sheffield, UK, revealing an inverse relationship between birth weight and systolic blood pressure, with a notable increment of 2.7 mmHg for every 450 g reduction in birth weight (Martyn et al., 1995). Further substantiating these findings, the Cardiovascular Risk in Young Finns study, which followed 3,596 subjects from childhood and adolescence (ages 3–18 years) through to middle adulthood (ages 34–49 years), demonstrated that IUGR-affected offspring exhibited subtle cardiac morphological alterations and diminished stroke volume. In a large-scale retrospective analysis, Leon et al. examined a cohort exceeding 15,000 births in Sweden, establishing a significant association between low birth weight and ischemic heart disease mortality in men aged 65 years and older, even after adjusting for socioeconomic variables (Leon et al., 1998). Collectively, these epidemiological investigations provide robust evidence that IUGR represents a common developmental antecedent for adult cardiovascular disease.

**FIGURE 2:**
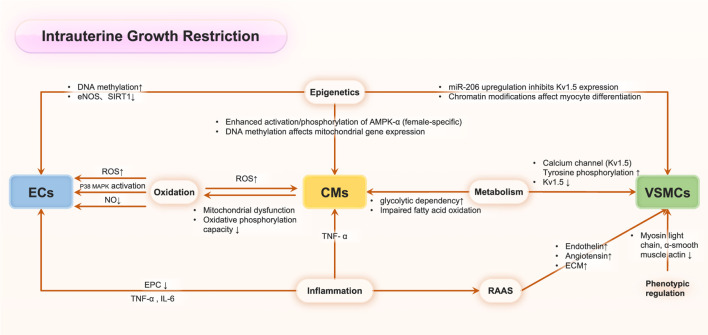
The molecular mechanisms in IUGR associated cardiovascular perturbations.

While the precise mechanisms underlying adult cardiovascular disease in IUGR remain to be fully elucidated, substantial evidence indicates that IUGR functions as a critical fetal programming factor in determining adult cardiovascular outcomes. IUGR has been demonstrated to elevate cardiovascular disease mortality rates in offspring and significantly impact cardiovascular health across generations (Anderson et al., 2006). During fetal development in IUGR conditions, adaptive responses to adverse uterine environments manifest as alterations in cardiac morphology, arterial remodeling, and modifications of all the cellular types involved in maintaining cardiovascular physiological function, including endothelial cells, fibroblast cells, cardiomyocytes and immune cells. For example, multiple researches demonstrated that IUGR offspring exhibited an increased risk of elevated blood pressure across multiple life stages: infancy (Masi et al., 2011), adolescence (Rossi et al., 2011; Nilsson et al., 1997), young adulthood (Leon et al., 2000), and later life (Martyn et al., 1995; Curhan et al., 1996; Law and Shiell, 1996), with notable sex-specific variations. Moreover, an emerging evidence had established a significant correlation between IUGR and adult coronary heart disease. Also, the potential mechanism had been explored, and Canoy et al. demonstrated an inverse relationship between IUGR and adult leukocyte count, suggesting an inflammatory mechanism potentially linking IUGR to adult coronary heart disease risk (Canoy et al., 2009). Alterations in coronary artery pathophysiology not only enhance coronary artery disease risk but also compromise myocardial perfusion in adulthood, serving as a significant risk factor for cardiac disorders. IUGR offspring demonstrate increased susceptibility to heart failure from various etiologies. Echocardiographic studies by Crispi and Carlos et al. in IUGR rat offspring revealed significantly elevated left ventricular end-diastolic diameter (LVEDD) and end-systolic diameter (LVESD), accompanied by reduced ejection fraction (Menendez-Castro et al., 2014; Crispi et al., 2010). Comparable findings were observed in an IUGR model demonstrating increased LVEDD and diminished ejection fraction in young rat offspring (Cheema et al., 2005), collectively indicating impaired contractile function with cardiomegaly. Coutinho et al. found that globular cardiac remodeling with enhanced systolic function but impaired diastolic indices in late-onset IUGR fetuses. Postnatally, offspring maintained elevated systolic performance and cardiac output with reduced LV torsion, indicating persistent cardiovascular adaptation to IUGR (Crispi et al., 2010; Bahtiyar and Copel, 2008). During prenatal programming, IUGR associates with adaptations involving increased peripheral resistance but decreased coronary and cerebral resistance, preserving vital organ function (Crispi et al., 2010; Bahtiyar and Copel, 2008). While early compensatory mechanisms, including myocardial vascular modifications and cardiac structural-functional alterations, maintain cardiac output initially, IUGR may ultimately lead to compromised cardiac contractile function and heart failure (Tare et al., 2012; Severi et al., 2000; Tintu et al., 2009). Although cumulative evidence demonstrates a potential lifelong relationship between IUGR and adult cardiovascular diseases, the severity of IUGR significantly influences the degree of injury and subsequent adult outcomes. Graupner et al. reported that global and segmental longitudinal peak systolic strain and strain rate values showed no significant differences between late-onset small-for-gestational-age and control fetuses for both ventricles, suggesting that mild IUGR may not significantly affect myocardial deformation properties measured by two-dimensional speckle tracking echocardiography (Graupner et al., 2022). Therefore, elucidating the potential molecular mechanisms (Figure 2) underlying these persistent physiological impacts across decades remains critical for advancing our understanding and therapeutic approaches.

## Cellular dysfunction associated with Iugr

### Endothelial cells

IUGR precipitates long-lasting alterations in vascular endothelial function, potentially contributing to cardiovascular morbidity in later life. The endothelium, serving as a crucial interface between circulating blood and vessel wall, plays a pivotal role in maintaining vascular homeostasis through the regulation of vascular tone, inflammation, and angiogenesis. IUGR-induced endothelial dysfunction manifests through multiple pathophysiological mechanisms. At the cellular level, IUGR significantly impacts endothelial progenitor cells (EPCs), crucial mediators of vascular repair and neoangiogenesis, by reducing their number and impairing their functionality (Peyter et al., 2021). This compromise in EPC biology is characterized by premature cellular senescence, altered proliferative capacity, and diminished angiogenic potential. Peyter and colleagues demonstrated that fetal developmental conditions significantly influence both the quantity and functionality of endothelial progenitor cells (EPCs) (Peyter et al., 2021; Peyter et al., 2014). Most studies indicate reduced EPC quantity and function in IUGR, regardless of the study models. Notably, Meister et al. documented a substantial 50% reduction in CD34^+^ cells among preterm neonates compared to their term counterparts. EPCs isolated from cord blood of low birth weight (LBW) newborns exhibited compromised angiogenic function, characterized by premature senescence mediated through diminished sirtuin 1 (SIRT1) activity. This dysfunction was further associated with endothelial microparticle release, triggered by the activation of mitogen-activated protein kinase six/p38 mitogen-activated protein kinase (MKK6/p38) signaling cascades, leading to heat shock protein 27 (Hsp27) phosphorylation, ultimately contributing to endothelial homeostatic disruption. At the molecular level, IUGR triggers complex cascades involving oxidative stress, inflammatory mediators, and alterations in nitric oxide (NO) signaling pathways (Rock et al., 2021). The dysregulation of these pathways leads to impaired endothelium-dependent vasodilation, enhanced vasoconstrictor responses, and structural remodeling of the vessel wall. The endothelial dysfunction associated with IUGR demonstrates remarkable persistence, extending from fetal life through adulthood, suggesting epigenetic modifications that program long-term vascular phenotypes. Due to the persistent oxidative stress and metabolic disorders from IUGR, endothelial colony-forming cells displayed a decreased proportion of CD31^+^ *versus* CD146+ staining on CD45^−^cells, CD34 expression, reduced proliferation, and an impaired capacity to form capillary-like structures, associated with an impaired angiogenic profile (Simoncini et al., 2021). This programming effect exhibits notable sex-specific differences, with male offspring often displaying more severe endothelial impairment. Understanding these mechanisms is crucial for developing targeted therapeutic strategies to prevent or ameliorate the cardiovascular sequelae of IUGR in affected offspring.

### Smooth muscle cells

IUGR represents a significant developmental perturbation that induces profound alterations in vascular smooth muscle cell (VSMC) structure and function, contributing to long-term cardiovascular complications in offspring. This pathological condition, affecting approximately 5%–10% of pregnancies globally, initiates a complex cascade of adaptive responses in VSMCs that persist well beyond the fetal period, suggesting substantial developmental programming effects on vascular smooth muscle phenotype and functionality. The impact of IUGR on VSMC function manifests through multiple mechanistic pathways. At the cellular level, IUGR induces significant alterations in VSMC phenotypic modulation, characterized by enhanced synthetic capacity and modified contractile protein expression patterns. These changes are accompanied by altered calcium handling mechanisms, increased myogenic tone, and enhanced contractile responses to vasoactive agents. Notably, IUGR-affected VSMCs demonstrate accelerated differentiation and heightened proliferative capacity, potentially contributing to vascular wall thickening and altered vessel compliance. Christie and colleagues demonstrated that IUGR significantly impacts calcium-dependent force generation in VSMCs. Their investigations revealed diminished peak responsiveness to potassium-induced depolarization, attributed to reduced maximum calcium-activated force in 6-month-old IUGR offspring (Fu et al., 2017; Lv et al., 2019). Furthermore, their findings indicated sex-specific alterations, with male IUGR rats exhibiting decreased abundance of critical receptor and contractile proteins, while females showed no significant changes. This sexual dimorphism in mesenteric artery reactivity was primarily attributed to reduced maximum calcium-activated force and diminished contractile protein and receptor/channel content in male rats at 6 months of age (Jiang et al., 2014; Christie et al., 2018). Subsequent molecular investigations by Fu et al. (Fu et al., 2017). Revealed enhanced tyrosine phosphorylation in pulmonary artery smooth muscle cells, leading to hyper-tyrosine phosphorylated voltage-gated potassium channel 1.5 (Kv1.5) with decreased expression, ultimately resulting in exacerbated adult vascular dysfunction. At the molecular level, IUGR induces epigenetic modifications in VSMCs, affecting key regulatory pathways involved in cell differentiation, proliferation, and contractility. Notably, VSMCs from 12-week-old IUGR rats demonstrated decreased right ventricular systolic pressure and reduced cell proliferation following chronic hypoxia exposure. Additionally, Lv and colleagues documented elevated microRNA-206 (miR-206) expression in resistance pulmonary arteries of 12-week-old IUGR rats compared to newborns (Lv et al., 2019). Significantly, *in vivo* or *in vitro* administration of miR-206 inhibitor resulted in increased expression of both Kv1.5 α-protein and KCNA5, suggesting a potential therapeutic target.

### Cardiomyocytes

IUGR represents a critical developmental perturbation that significantly impacts cardiac development and cardiomyocyte function, potentially programming long-term cardiovascular vulnerability in offspring (Alexander et al., 2015; Alici Davutoglu et al., 2020). This pathological condition, characterized by impaired fetal growth and development, induces substantial alterations in cardiomyocyte structure, metabolism, and functional capacity that persist into postnatal life. At the cellular level, IUGR profoundly affects cardiomyocyte development and maturation through multiple mechanisms (Jonker et al., 2018). Notable alterations include reduced cardiomyocyte proliferation, premature terminal differentiation, and accelerated cellular aging. These changes result in a reduced total number of cardiomyocytes at birth, potentially compromising cardiac functional reserve throughout life (Vranas et al., 2017). Furthermore, IUGR-affected cardiomyocytes exhibit distinct morphological changes, including altered cell size, modified sarcomeric organization, and disrupted mitochondrial architecture (Akazawa et al., 2016). Recent investigations utilizing non-human primate models have provided valuable insights into the translational aspects of IUGR-induced cardiac dysfunction. Kuo and colleagues developed a baboon model of IUGR through moderate maternal caloric restriction (30%), bridging the gap between rodent models and human pathophysiology (Kuo et al., 2017a; Kuo et al., 2017b). Cardiac magnetic resonance imaging (MRI) of IUGR offspring revealed impaired diastolic and systolic function, characteristic of premature cardiac aging (Kuo et al., 2017b). Additionally, right ventricular filling and ejection abnormalities were observed in young adult IUGR baboons, suggesting comprehensive cardiac dysfunction. Sex-specific cardiac vulnerability has been demonstrated in rodent models, particularly in response to intrauterine hypoxia. Boltting and colleagues reported that maternal hypoxia specifically reduced cardiomyocyte numbers in female offspring, while male offspring-maintained cardiomyocyte populations (Botting et al., 2018). Intriguingly, maternal nutrient restriction did not affect cardiomyocyte numbers in either sex. At the molecular level, male IUGR offspring exhibited decreased expression of genes governing fatty acid activation in the sarcoplasm and mitochondrial transport. In contrast, females exposed to maternal hypoxia demonstrated enhanced activation/phosphorylation of AMP-activated protein kinase-α (AMPK-α). These findings suggest that alterations in cardiomyocyte endowment and cardiac gene expression profiles are direct consequences of *in utero* metabolic programming, with distinct sex-specific patterns.

## The molecular mechanisms In adulthood Cvd post IUGR

### Postnatal catch-up growth features

The complex interplay between prenatal growth restriction and postnatal adaptation mechanisms significantly influences organ development and long-term health outcomes. While compensatory catch-up growth is frequently observed during the postnatal period in IUGR offspring, this adaptive response may paradoxically contribute to adverse cardiometabolic consequences. Nevin and colleagues demonstrated that post-IUGR catch-up growth exhibits marked organ-specific heterogeneity during early life, with differential growth trajectories persisting into adulthood (Nevin et al., 2018). Notably, cardiac tissue showed minimal compensatory growth response postnatally, potentially predisposing to structural abnormalities and elevated disease risk in later life. In experimental models, IUGR offspring manifest significant cardiovascular perturbations by 4 months of age, characterized by hypertension, increased abdominal adiposity, and elevated circulating leptin and total cholesterol concentrations (Chu et al., 2019). These findings establish a mechanistic link between placental transcriptional responses to gestational hypoxia and subsequent cardiometabolic disease susceptibility (Kumar et al., 2020; Lane et al., 2020; Verschuren et al., 2012; Xu et al., 2013). Recent investigations by Yuliana and colleagues revealed that IUGR secondary to uteroplacental insufficiency induces significant pulmonary developmental impairment, characterized by reduced alveolar space, decreased proliferating cell nuclear antigen (PCNA) expression, and increased alveolar wall volume fraction, concurrent with diminished leptin expression (Yuliana et al., 2024). These findings suggest therapeutic potential for leptin supplementation in IUGR-associated pulmonary complications (Yan et al., 2019). The metabolic consequences of IUGR followed by accelerated postnatal growth are particularly noteworthy. Berends and colleagues demonstrated that IUGR mice experiencing catch-up growth develop both peripheral and central insulin resistance in adulthood (Berends et al., 2018). Mechanistically, this central insulin resistance was associated with reduced protein levels of the p110β subunit of phosphoinositide 3-kinase (PI3K) and enhanced serine phosphorylation of insulin receptor substrate-1 (IRS-1) in the hypothalamic arcuate nucleus (ARC). These molecular alterations may underlie the resistance to insulin’s anorectic effects and impaired glucose homeostasis observed in these animals. Significant advances in therapeutic interventions have emerged from recent research. Alsaied and colleagues demonstrated that IUGR offspring exhibit significantly impaired systolic cardiac function, manifesting as reduced fractional shortening compared to controls (Alsaied et al., 2017). Notably, intraplacental IGF-1 administration following uterine artery ligation showed promising results in improving cardiac function and preventing adult cardiovascular disease, suggesting a potential therapeutic strategy for IUGR-associated cardiac dysfunction.

### ECM remodeling

IUGR induces profound alterations in cardiovascular structure and function, with particular emphasis on extracellular matrix (ECM) remodeling and vascular compliance (Dodson et al., 2013; Mankouski et al., 2022). The long-term risk of hypertension associated with dietary factors is significantly amplified by IUGR-induced structural modifications to the ECM, characterized by persistent alterations in elastin composition and increased arterial stiffness. Furthermore, IUGR significantly impairs the differentiation potential of mesenchymal stem cells (MSCs), manifesting as enhanced adipogenic capacity through upregulated expression of peroxisome proliferator-activated receptor gamma (PPARγ) and fatty acid-binding protein 4 (FABP4), concurrent with increased fibrogenic potential evidenced by elevated collagen type I alpha 1 (COL1A1) expression (Weatherall et al., 2020). Mankouski and colleagues demonstrated that IUGR promotes early-life vascular ECM remodeling and increased vascular stiffness, leading to elevated blood pressure (Mankouski et al., 2022). Their findings also revealed that excessive caloric intake in IUGR subjects resulted in dyslipidemia and increased mortality rates. Mechanistic insights from Li and colleagues identified enhanced activation of the Notch1 signaling pathway in IUGR-affected human umbilical vein endothelial cells (HUVECs), with subsequent activation of protein kinase B (Akt) and extracellular signal-regulated protein kinases 1/2 (ERK1/2) (Li M. et al., 2022). Notably, both pharmacological inhibition and genetic silencing of Notch1 attenuated the proliferative phenotype of IUGR-induced HUVECs and reduced ERK1/2 and AKT activation. Comprehensive proteomic and bioinformatic analyses by Zouridis and colleagues revealed that IUGR significantly downregulates integrin signaling components (ITGA1, ITGB3, and ILK) and apelin cardiac fibroblast signaling pathways (Zouridis et al., 2021). Additionally, they observed decreased muscle contraction and glycolytic enzyme expression (PKM, PFKL, and PFKP), alongside overrepresentation of protein networks associated with embryonic development (MYH7) and cell survival pathways. Hypoxia-induced IUGR has been associated with nuclear morphological alterations and increased collagen fiber thickness in early postnatal offspring, suggesting premature fibrosis in arterial walls correlating with increased aortic stiffness (Kumar et al., 2020). These structural changes are mechanistically linked to progressive left ventricular diastolic dysfunction secondary to placental insufficiency. Furthermore, IUGR significantly impairs aortic development, followed by compensatory hypertrophic remodeling during lactation. Gutiérrez-Arzapalo and colleagues demonstrated that fetal undernutrition results in compromised aortic development, subsequently leading to hypertrophic remodeling and enhanced aortic compliance during the perinatal period, with corresponding alterations in collagen and elastin composition (Gutiérrez-Arzapalo et al., 2018). Notably, aortic elastin scaffolds isolated from young IUGR animals of both sexes exhibited increased compliance, though this phenotype persisted only in adult IUGR females. This sexual dimorphism in vascular adaptation appears to serve as a compensatory mechanism preventing hypertension development, with the differential accumulation of collagen and elastin in female offspring conferring a protective effect absent in male counterparts.

### eNOS

Endothelial dysfunction represents a critical pathophysiological mechanism underlying cardiovascular complications associated with IUGR. The complex interplay between oxidative stress, vasoactive mediators, and endothelial signaling pathways significantly influences vascular function and long-term cardiovascular outcomes in IUGR offspring (Wang et al., 2023; Guillot et al., 2023; Sutovska et al., 2022; Wohlmuth et al., 2020; Krause et al., 2019). Understanding these molecular mechanisms is crucial for developing targeted therapeutic strategies to prevent programmed cardiovascular disease. Experimental studies utilizing eNOS knockout mice have demonstrated that IUGR induces multiple adverse outcomes consistent with programmed cardiometabolic disease. Notably, pharmacological intervention with sildenafil enhanced acetylcholine-mediated vasodilation in mesenteric arteries, suggesting potential therapeutic applications (Mills et al., 2018). Guillot and colleagues elucidated that IUGR-associated arterial hypertension stems from oxidative stress-induced premature senescence, characterized by impaired vasoconstriction regulation (Guillot et al., 2023). Their molecular analyses revealed attenuated expression of eNOS, copper/zinc superoxide dismutase (Cu/Zn SOD), and SIRT1, concurrent with elevated p16 levels. Importantly, early administration of antioxidant polyphenol compounds demonstrated the capacity to reverse IUGR-induced oxidative damage and preserve arterial function. Selivanova and colleagues reported enhanced contractile responses to thromboxane A2 receptor agonist U46619 in coronary arteries of IUGR rats, attributed to diminished NO-mediated anticontractile effects and increased Rho-kinase activity in the endothelium (Selivanova et al., 2021). These functional alterations were accompanied by reduced SOD1 protein expression and elevated RhoA protein content in coronary vessels of IUGR subjects.

IUGR significantly impacts perinatal morbidity and mortality, with profound implications for long-term cardiovascular health. He and colleagues identified that disrupted ET-1 and endothelin B receptor (ET(B)R) signaling contributes to reduce NO bioavailability and enhanced arginase-2 (Arg-2) release, promoting ADMA accumulation and subsequent vascular endothelial dysfunction (He et al., 2018). These findings provide novel mechanistic insights into fetal programming associated with IUGR, potentially identifying therapeutic targets for preventing adverse cardiovascular outcomes in adulthood. Rock and colleagues demonstrated that IUGR-induced alterations in resistance artery function involve multiple vasoactive pathways, including NO, prostanoids, and endothelium-dependent hyperpolarization (Rock et al., 2021). Their investigations revealed a progressive decline in endothelium-dependent vasodilation in IUGR lambs compared to controls, with specific impairment of the prostanoid pathway emerging as a key contributor to reduced vasodilatory capacity.

### RAAS

IUGR fundamentally alters cardiovascular development through complex interactions between hemodynamic adaptations, molecular signaling pathways, and structural remodeling (Sehgal et al., 2023). The pathophysiological cascade initiated by IUGR encompasses both immediate adaptations and long-term programmed changes in cardiovascular structure and function, with particular emphasis on the renin-angiotensin-aldosterone system (RAAS) and its downstream effects on vascular architecture and renal function (Sehgal et al., 2020). Impaired uteroplacental perfusion and consequent fetal hypoxemia trigger significant alterations in arterial structure and function. These changes are characterized by a decreased arterial wall elastin-to-collagen ratio, compromised endothelial function, and heightened RAAS activation. The amplified RAAS signaling cascade induces increased expression of pro-inflammatory molecules, notably monocyte chemoattractant protein-1 (MCP-1), while simultaneously disrupting matrix structure through elevated matrix metalloproteinase-2 (MMP-2) and transforming growth factor-beta 1 (TGF-β1) expression (Sehgal et al., 2023). Concurrent attenuation of cytoskeletal proteins, including desmin and vimentin, further contributes to increased arterial wall stiffness, as demonstrated by Sehgal and colleagues.

Voggel and colleagues revealed that IUGR-induced hypertension is intricately linked to impaired kidney function, with IUGR rat offspring exhibiting enhanced vulnerability to hypertensive glomerular damage (Voggel et al., 2021). This susceptibility is attributed to diminished myogenic tone and paradoxically augmented endothelium-dependent relaxation in renal interlobar arteries (RIAs). Chen and colleagues demonstrated that IUGR offspring exposed to an 8% high-salt diet developed significant cardiac hypertrophy, whereas ouabain treatment ameliorated these adverse effects (Chen et al., 2015). Their findings suggest that ouabain’s beneficial effects, including restoration of glomerular number in neonates and normalization of blood pressure in adult male IUGR offspring, can preserve cardiac structure and function, particularly under high-salt dietary challenges. These observations underscore the critical importance of RAAS normalization in managing IUGR-associated programmed cardiovascular pathological compensations. Black and colleagues made the notable observation that female IUGR subjects lose their protective adaptation against age-related decline in renal and arterial function, manifesting as elevated arterial pressure without apparent renal structural damage (Black et al., 2015). This sexual dimorphism in cardiovascular adaptation highlights the complex interaction between IUGR and renal signaling regulation, particularly in the context of RAAS dysfunction.

### Oxidative stress and mitochondrial damages

Metabolic perturbations and oxidative stress represent fundamental mechanisms underlying cardiovascular dysfunction in IUGR (Li P. et al., 2022; Rasmussen et al., 2022; Pereira et al., 2023; Dimasi et al., 2023). These alterations encompass mitochondrial dysfunction, altered energy metabolism, and disrupted redox homeostasis, which collectively contribute to programmed cardiovascular disease. The complex interplay between these metabolic disturbances and cardiovascular function demonstrates significant sexual dimorphism and presents potential therapeutic targets for intervention. Metabolic dysregulation manifests prominently in IUGR offspring, characterized by accumulated reactive oxygen species (ROS) due to enhanced NADPH oxidase expression and diminished antioxidant capacity, particularly reduced copper/zinc superoxide dismutase in arterial walls (von Ehr and von Versen-Höynck, 2016; Kimura et al., 2013). Sehgal and colleagues interpreted these alterations as indicative of accelerated arterial aging, ultimately contributing to hypertension development (Sehgal et al., 2023; Sehgal et al., 2020). Reyes and colleagues demonstrated that cardiomyocytes from prenatally hypoxic male offspring exhibited decreased proliferation compared to controls, attributed to oxidative stress-mediated effects (Reyes et al., 2018). Notably, administration of recombinant TNF-like weak inducer of apoptosis (r-TWEAK) enhanced cardiomyocyte proliferation across all offspring groups. Pereira and colleagues unveiled persistent mitochondrial impairment in IUGR cardiomyocytes, with protein diet restriction-induced IUGR resulting in sex-specific alterations in oxidative phosphorylation (OXPHOS) subunit gene expression, predominantly affecting complexes I, III, and ATP synthase, with more pronounced effects in female subjects (Pereira et al., 2023; Pereira et al., 2021). These findings suggest that early IUGR-induced mitochondrial adaptations contribute to offspring mitochondrial dysfunction and increased cardiovascular disease susceptibility in a sex-dependent manner. Spiroski and colleagues identified that IUGR induced NADPH-mediated hypoxic damage, manifesting as increased α_1_-adrenergic cardiovascular reactivity, impaired peripheral vascular reactive hyperemia, and cardiac alterations including sympathetic dominance, hypercontractility, and diastolic dysfunction (Spiroski et al., 2021). Importantly, mitochondrial antioxidant therapy demonstrated protective effects against NADPH-induced oxidative damage, potentially preventing programmed cardiovascular disease in adult offspring of hypoxic pregnancies. Dimasi and colleagues reported reduced myocardial redox ratios in IUGR subjects, indicating increased reliance on glycolytic ATP production. They established a positive correlation between left ventricular cardiac output (LVCO) and redox ratio in IUGR, suggesting that altered cardiac metabolism may contribute to reduced cardiac output and subsequent poor cardiovascular outcomes in adulthood (Dimasi et al., 2023; Dimasi and Darby, 2022; Dimasi et al., 2021). Guitart-Mampel and colleagues demonstrated significant alterations in cord blood mononuclear cells (CBMC) from IUGR patients, characterized by decreased mitochondrial respiratory chain complex I enzymatic activity and enhanced oxygen consumption, accompanied by elevated SIRT3/β-actin protein levels (Guitart-Mampel et al., 2019). Additionally, they observed reduced citrate synthase activity in IUGR newborns, although total ATP levels and lipid peroxidation remained unchanged.

### mtDNA

IUGR represents a significant developmental challenge that programs increased cardiometabolic risk through complex pathophysiological mechanisms. These mechanisms encompass alterations in mitochondrial function, inflammatory signaling pathways, and endothelial dysfunction, collectively contributing to the development of cardiovascular complications in later life. The interaction between these pathways demonstrates the intricate relationship between early life adversity and long-term cardiovascular health outcomes. Whereas, mitochondrial DNA (mtDNA) had been demonstrated as a critical mediator in regulating cardiovascular function, especially in the interplays between inflammation and cardiac injuries via mitophagy signaling (Pereira et al., 2021; Iodice et al., 2018; Oliveira et al., 2017a).

Substantial evidence has emerged linking IUGR to chronic low-grade inflammation, which serves as a critical mediator in the development of various cardiometabolic comorbidities. Oliveira and colleagues conducted pioneering research demonstrating that IUGR programs alterations in circulating mitochondrial DNA (mtDNA)/Toll-like receptor 9 (TLR9) content, leading to elevated tumor necrosis factor-alpha (TNF-α) levels and a persistent pro-inflammatory state (Oliveira et al., 2017a). Notably, their research revealed that aerobic training could effectively normalize these inflammatory perturbations, suggesting a potential therapeutic intervention strategy. In a subsequent pilot study, Oliveira and colleagues investigated post-IUGR endothelial function, revealing significant impairments in acetylcholine-mediated vasodilation in aortic rings, accompanied by reduced NO levels and enhanced eNOS phosphorylation at threonine 495 (Oliveira et al., 2017b). Furthermore, IUGR was found to compromise the pluripotency of bone marrow-derived endothelial progenitor cells, contributing to sustained endothelial dysfunction. Pereira and colleagues provided crucial insights into the molecular mechanisms underlying IUGR-induced cardiac dysfunction (Pereira et al., 2023). Their investigation revealed that maternal nutrient restriction-induced IUGR resulted in increased fetal left ventricular mitochondrial DNA content and elevated levels of key respiratory chain proteins, including NDUFB8, UQCRC1, and cytochrome c (Pereira et al., 2021). However, despite these compensatory increases, overall mitochondrial metabolic function was compromised, characterized by reduced complex I and complex II/III activities. These findings suggest that IUGR initiates a developmental programming cascade leading to adult cardiac dysfunction, primarily through programmed mitochondrial inefficiency in energy delivery to cardiac tissues, potentially serving as a fundamental mechanism for heart failure development in later life.

### Fatty acid and glucose metabolism

Metabolic adaptations in IUGR involve complex alterations in substrate utilization, particularly affecting lipid metabolism and glucose homeostasis (Sehgal et al., 2023; Küster et al., 2023). These metabolic perturbations, resulting from uteroplacental insufficiency, manifest through multiple pathways including altered fatty acid metabolism, compromised mitochondrial function, and modified insulin signaling. Understanding these adaptations is crucial for developing targeted therapeutic strategies for IUGR-associated cardiovascular complications. Uteroplacental insufficiency-induced IUGR offspring exhibit significant reductions in vessel density accompanied by altered acylcarnitine profiles, indicating impaired mitochondrial lipid metabolism. Drake and colleagues demonstrated that IUGR fetuses present with elevated circulating long-chain fatty acylcarnitines compared to controls, suggesting compromised fatty acid metabolism (Drake et al., 2022). Their innovative approach utilizing fluorescently tagged BODIPY-C12-saturated free fatty acid in live, isolated cardiomyocytes revealed that while lipid droplet morphometry (area and number) remained unchanged between control and IUGR cells, significant molecular alterations were evident. Specifically, they observed widespread suppression of key metabolic genes in IUGR myocardium (P < 0.05), including sarcolemmal fatty acid transporters (CD36, FATP6), acylation enzymes (ACSL1, ACSL3), mitochondrial transporter (CPT1), β-oxidation enzymes (LCAD, HADH, ACAT1), tricarboxylic acid cycle enzyme (IDH), esterification enzymes (PAP, DGAT), and the lipid droplet formation regulator (BSCL2) (Dimasi et al., 2023; Drake et al., 2022; Abbasi et al., 2012). Yates and colleagues provided compelling evidence for IUGR-induced alterations in insulin sensitivity and glucose metabolism (Yates et al., 2019). Their investigations in one-month-old IUGR lambs revealed enhanced rates of hindlimb glucose uptake, potentially serving as a compensatory mechanism for myocyte deficiencies in glucose oxidation, characterized by reduced GLUT4 expression. The impairment in glucose-stimulated insulin secretion observed in IUGR lambs was attributed to decreased intra-islet insulin availability rather than defective glucose sensing mechanisms. Furthermore, their analysis of adrenergic receptor β2 (ADRβ2) desensitization revealed that ADRβ2 activation enhanced whole-body glucose utilization rates and insulin sensitivity (Yates et al., 2019). Notably, selective ADRβ2 stimulation combined with β1/β3 inhibition resulted in improved insulin sensitivity and increased whole-body glucose utilization in IUGR lambs.

### Persist inflammation attacks

IUGR disrupts cardiovascular and immune systems via multifaceted mechanisms, programming lifelong dysfunction. The developmental origins of health and disease hypothesis suggests that adverse events during critical periods of fetal development can permanently alter organ structure and function, leading to increased disease susceptibility throughout life. Recent advances in molecular biology techniques, including transcriptome sequencing and single-cell RNA sequencing, have provided unprecedented insights into the mechanisms underlying these programmed alterations. IUGR exerts substantial adverse effects on long-term cardiovascular function in experimental rat models, as evidenced by programmed metabolic dysfunction and chronic inflammatory activity identified through transcriptome sequencing in IUGR offspring (Li P. et al., 2022). These early-life adverse events encode lifelong disease risk, which may remain subclinical during youth but manifest later in life. This understanding emphasizes the importance of investigating long-term molecular alterations, as nutritional intervention alone proves insufficient to fully reverse IUGR-induced damage. Visentin and colleagues demonstrated significantly reduced adiponectin levels in IUGR subjects, accompanied by elevated leptin, TNFα, and IL-6 levels (Visentin et al., 2014a; Visentin et al., 2014b; Visentin et al., 2013). Notably, IUGR fetuses exhibited increased aortic intima-media thickness, strongly correlating with reduced circulating adiponectin and various inflammatory markers, suggesting a potential causal relationship between persistent inflammation and vascular remodeling (Visentin et al., 2013).

In maternal protein restriction-induced IUGR offspring, researchers observed sustained upregulation of TNF-α expression and JNK phosphorylation in hepatic tissue from fetal development through adulthood. Elevated JNK phosphorylation was associated with downregulated hepatocyte nuclear factor-4α and CYP7A1 expression, leading to increased hepatic cholesterol and enhanced metabolic syndrome risk (Liu et al., 2014). Yamauchi and colleagues demonstrated that AT2R knockout exacerbated pathological manifestations associated with low-protein diet-induced IUGR, resulting in increased left ventricular wall thickness, enlarged cardiomyocyte size, and enhanced perivascular fibrosis (Yamauchi et al., 2018). Notably, collagen I and inflammatory cytokine mRNA expression were significantly elevated in AT2R knockout IUGR offspring hearts at 6 weeks but not at 12 weeks of age. Ushida and colleagues provided compelling evidence for transgenerational inheritance of cardiometabolic alterations in IUGR subjects (Ushida et al., 2022a). The aberrant inflammation associated with IUGR compromised cardiometabolic status in F1 offspring, increasing susceptibility to infection and other adverse exposures. The F1 generation exhibited enhanced macrophage infiltration in placental tissue and reduced glucose transporter-1 expression in uteroplacental units (Ushida et al., 2022b). Pecks and colleagues demonstrated reduced cholesterol efflux capacity in IUGR compared to controls, strongly correlating with HDL and apoA1 levels, potentially contributing to enhanced cardiovascular disease risk later in life (Pecks et al., 2019).

Advanced single-cell RNA sequencing (scRNA-seq) analysis has revolutionized our understanding of cellular heterogeneity in IUGR. Bacon and colleagues utilized scRNA-seq to investigate thymic maturation in growth-restricted neonatal mice, revealing significant heterogeneity among CD8/CD4 double-positive cells, with one subcluster showing marked upregulation of ribosomal surface protein transcripts (Bacon et al., 2018). Their findings indicated skewed cellular distribution in IUGR animals, characterized by increased immature CD8/CD4 double-negative cells and reduced mature T-cells. This T-cell deficit persisted into adulthood despite catch-up growth normalizing body and organ weights, suggesting potential long-term implications for adult immunity and adding to the growing list of adult conditions influenced by the intrauterine environment.

### Epigenetic modification

Epigenetic modifications represent crucial molecular mechanisms through which IUGR programs long-term cardiovascular dysfunction. These modifications, including DNA methylation, histone modifications, and non-coding RNAs, can persist throughout life and significantly influence gene expression patterns in cardiovascular tissues. Recent technological advances in epigenome-wide analysis have enabled comprehensive investigation of these modifications and their functional consequences in cardiovascular development and disease. High-resolution DNA methylation array analysis has revealed differentially methylated regions in the promoters of eight critical genes: DECR1, ZNF300, DNAJA4, CCL28, LEPR, HSPA1A/L, GSTO1, and GNE, implicating aberrations in fatty acid beta-oxidation and transcriptional regulation in IUGR offspring (Roifman et al., 2016). Terstappen and colleagues demonstrated that IUGR compromises offspring endothelial function through downregulation of LGALS1, FPR3, and NRM, concurrent with upregulation of lincRNA RP5-855F14.1 (Terstappen et al., 2020). Notably, male offspring exhibited specific DNA methylation changes in FPR3 and NRM genes, correlating with cardiovascular remodeling and pathology (Terstappen et al., 2020). Yan and colleagues reported that IUGR and associated catch-up growth significantly impair pulmonary vascular development in rat models (Yan et al., 2019). Their findings revealed decreased mRNA expression of the memory-related gene zif268 and transcription factor recruitment factor p300 during catch-up growth, suggesting epigenetically regulated pulmonary arteriolar remodeling and eNOS expression. Paz and colleagues demonstrated that IUGR induces premature vascular aging in offspring, characterized by initially preserved carotid artery reactivity followed by significant impairment in NO-mediated responses (Paz et al., 2019). Femoral arteries from IUGR fetuses exhibited increased contractility but decreased relaxation responses compared to controls, with both parameters deteriorating in IUGR adults. Mechanistically, decreased LINE-1 DNA methylation in endothelial cells mediated vascular function in a NO-dependent manner, while reduced eNOS mRNA levels in IUGR adults correlated with increased DNA methylation (Paz et al., 2019).

In the context of diabetic pregnancy, Golic and colleagues identified alterations in cholesterol metabolism mediated through epigenetic modification of Srebf2 (sterol regulatory element binding transcription factor 2) (Golic et al., 2018). They observed CpG hypermethylation in the Srebf2 promoter region, suggesting this transcription factor as a potential mediator of intrauterine environment-driven epigenetic changes affecting offspring health. Paules and colleagues demonstrated that IUGR induces premature aging in vascular endothelium, characterized by elevated caspase-3 and p53 levels, shortened telomeres, and reduced SIRT1 expression (Paules et al., 2019). These parameters showed significant linear trends correlating with disease severity. Tang and colleagues revealed decreased pulmonary vascular density associated with reduced Notch1 transcription and its downstream target Hes-1 at both 3 and 9 weeks (Tang et al., 2015). Their chromatin immunoprecipitation and bisulfite sequencing analyses demonstrated IUGR-induced increases in H3K27me3 in the proximal Notch1 gene promoter and enhanced methylation of specific CpG sites in the distal promoter region, persisting from 3 to 9 weeks.

These epigenetic modifications established during fetal development create a molecular memory that influences cardiovascular health throughout life, potentially contributing to programmed cardiovascular diseases in adulthood. Understanding these mechanisms is crucial for developing targeted therapeutic strategies to prevent or mitigate IUGR-associated cardiovascular complications.

### Potential therapeutic approaches

Therapeutic interventions for intrauterine growth restriction (IUGR) represent a critical area of cardiovascular research, as early-life interventions may potentially mitigate long-term cardiovascular complications. Current therapeutic strategies primarily focus on four major approaches: attenuation of hypoxic conditions, antioxidant supplementation, exercise intervention, and gene therapy. Each approach targets distinct pathophysiological mechanisms underlying IUGR-induced cardiovascular dysfunction.

### Hypoxia attenuation strategies

Melatonin has emerged as a promising therapeutic agent for protecting placental function during adverse pregnancy conditions. Itani and colleagues demonstrated that melatonin administration in hypoxic chick embryos effectively rescued cardiac systolic dysfunction, improved cardiac contractility and relaxability, reduced cardiac sympathetic dominance, and restored endothelial function in peripheral circulations (Itani et al., 2016). Using established rodent model of hypoxic pregnancy, Hansell et al. confirmed that maternal melatonin treatment can prevent cardiovascular dysfunction caused by hypoxia in offspring and normalize cardiac structure, eNOS expression and vascular reactivity (Hansell et al., 2022). The therapeutic mechanisms involved reduction of oxidative stress, enhancement of antioxidant capacity, restoration of VEGF expression, and improved NO bioavailability. Terstappen and colleagues corroborated these findings in animal studies, demonstrating that sildenafil citrate (60 mg/kg daily) enhanced endothelium-dependent and independent relaxation in treated male offspring (Terstappen et al., 2019). In a landmark double-blind randomized placebo-controlled trial, Shehata and colleagues evaluated sildenafil citrate’s efficacy in treating severe IUGR (Shehata et al., 2020). The intervention group showed significant improvements in umbilical artery pulsatility index and middle cerebral artery pulsatility index, suggesting enhanced uteroplacental and fetal cerebral perfusion. Subsequent studies have also confirmed that sildenafil increases birth weight and pregnancy elongation (Rakhanova et al., 2023; Liu et al., 2024). Fasudil, a Rho kinase inhibitor, has demonstrated promising cardiovascular effects through multiple mechanisms (Butruille et al., 2012). Zhou and colleagues revealed fasudil’s ability to enhance microcirculation through increased myosin light chain phosphatase activity (Zhou et al., 2019). Ojeda and colleagues demonstrated fasudil’s effectiveness in attenuating hemodynamic changes and reducing blood pressure responses to acute Angiotensin II in gonadectomized male IUGR offspring (Ojeda et al., 2013). Butruille and colleagues further showed that prenatal fasudil administration increased maternal hypothalamic NPY orexigenic gene expression, promoting dietary intake and ameliorating fetal growth in IUGR rats (Butruille et al., 2012).

### Antioxidant interventions

Oxidative stress plays a central role in IUGR-induced cardiovascular programming. Shah and colleagues demonstrated sex-specific benefits of resveratrol treatment in adult IUGR offspring, with distinct mechanisms between males and females (Shah et al., 2017). Notably, resveratrol increased cardiac p-AMPK and SOD2 levels exclusively in female IUGR offspring. Lane and colleagues investigated the selective PPAR-γ agonist pioglitazone (PIO) for preventing hypoxia-induced IUGR, demonstrating partial prevention of fetal growth restriction and attenuation of placental insufficiency. Tarry-Adkins and colleagues showed that CoQ10 supplementation prevented liver fibrosis and reduced oxidative stress, inflammation, and hyperinsulinemia in IUGR offspring with accelerated postnatal catch-up growth (Tarry-Adkins et al., 2016).

### Exercise intervention

The efficacy of exercise intervention shows sex-specific responses. Reyes and colleagues demonstrated that aerobic exercise training improved endothelium-derived hyperpolarization-mediated vasodilation in male IUGR offspring’s gastrocnemius muscle arteries (Reyes et al., 2015). However, female IUGR offspring showed reduced NO-mediated vasodilation and increased prostaglandin-mediated vasoconstriction, with exercise training showing no beneficial effects. Notably, exercise actually exacerbated cardiac function in adult male offspring exposed to prenatal hypoxia, suggesting it may act as a secondary stressor increasing superoxide production.

### Gene therapy approaches

Carr and colleagues investigated adenoviral-based VEGF-A165 gene therapy, demonstrating sex-specific effects on postnatal development (Carr et al., 2016; Carr et al., 2014). While the therapy influenced postnatal weight growth in female offspring, maternal nutrient restriction reduced heart weight regardless of Ad. VEGF-A165 treatment. Male offspring showed no significant responses to either maternal nutrient restriction or Ad. VEGF-A165 therapy regarding postnatal weight gain, heart morphology, or blood pressure.

These diverse therapeutic approaches highlight the complexity of treating IUGR-induced cardiovascular dysfunction and emphasize the need for personalized, sex-specific interventional strategies. Future research should focus on optimizing timing, dosage, and combination therapies while considering sex-specific responses to different therapeutic modalities. Targeted gene editing (such as CRISPR-based epigenetic reprogramming) and extracellular vesicle therapy from stem cells may also be potential therapeutic modalities for IUGR.

## Conclusion

IUGR, resulting from diverse adverse intrauterine environmental exposures, represents a significant risk factor for cardiovascular disease development in offspring. The developmental origins of health and disease hypothesis posits that these early-life perturbations induce structural and functional cardiovascular alterations detectable in early postnatal life, which may persist into adulthood. This review synthesizes recent advances in understanding the molecular mechanisms underlying fetal programming of adult cardiovascular disease in IUGR offspring. Particular emphasis is placed on the complex interplay of epigenetic modifications, mitochondrial dysfunction, altered lipid metabolism, oxidative stress, endoplasmic reticulum stress, inflammatory signaling cascades, and dysregulated autophagy pathways. These mechanistic insights have revealed novel therapeutic targets and potential interventional strategies. Understanding these fundamental pathophysiological processes is crucial for developing targeted preventive measures and therapeutic interventions to improve cardiovascular outcomes in individuals with a history of IUGR. Furthermore, this mechanistic understanding may facilitate the identification of early biomarkers for risk stratification and the development of personalized therapeutic approaches for this vulnerable population. The reviewed studies presented several methodological limitations. The abundance of confounding variables in human investigations necessitated additional longitudinal cohort studies, standardization of IUGR models, and comprehensive stratified analyses that accounted for sex- and age-specific variations. Furthermore, subsequent research endeavors required the integration of multiomics profiling approaches, machine learning algorithms for risk stratification, and organoid-based modeling systems to elucidate IUGR-induced cardiovascular pathogenesis and facilitate the development of targeted therapeutic interventions.

## References

[B1] Abbasi A. Thamotharan M. Shin B. C. Jordan M. C. Roos K. P. Stahl A. (2012). Myocardial macronutrient transporter adaptations in the adult pregestational female intrauterine and postnatal growth-restricted offspring. Am. J. Physiol. Endocrinol. Metab. 302 (11), E1352–E1362. 10.1152/ajpendo.00539.2011 22338075 PMC3378069

[B2] Akazawa Y. Hachiya A. Yamazaki S. Kawasaki Y. Nakamura C. Takeuchi Y. (2016). Cardiovascular remodeling and dysfunction across a range of growth restriction severity in small for gestational age infants - implications for fetal programming. Circ. J. 80 (10), 2212–2220. 10.1253/circj.CJ-16-0352 27535477

[B3] Alexander B. T. Dasinger J. H. Intapad S. (2015). Fetal programming and cardiovascular pathology. Compr. Physiol. 5 (2), 997–1025. 10.1002/cphy.c140036 25880521 PMC4772789

[B4] Alici Davutoglu E. Ozel A. Oztunc F. Madazli R. (2020). Modified myocardial performance index and its prognostic significance for adverse perinatal outcome in early and late onset fetal growth restriction. J. Matern. Fetal Neonatal Med. 33 (2), 277–282. 10.1080/14767058.2018.1489534 30033784

[B5] Alsaied T. Omar K. James J. F. Hinton R. B. Crombleholme T. M. Habli M. (2017). Fetal origins of adult cardiac disease: a novel approach to prevent fetal growth restriction induced cardiac dysfunction using insulin like growth factor. Pediatr. Res. 81 (6), 919–925. 10.1038/pr.2017.18 28099426

[B6] Anderson C. M. Lopez F. Zimmer A. Benoit J. N. (2006). Placental insufficiency leads to developmental hypertension and mesenteric artery dysfunction in two generations of Sprague-Dawley rat offspring. Biol. Reprod. 74 (3), 538–544. 10.1095/biolreprod.105.045807 16306423

[B7] Bacon W. A. Hamilton R. S. Yu Z. Kieckbusch J. Hawkes D. Krzak A. M. (2018). Single-cell analysis identifies thymic maturation delay in growth-restricted neonatal mice. Front. Immunol. 9, 2523. 10.3389/fimmu.2018.02523 30443254 PMC6221967

[B8] Bahtiyar M. O. Copel J. A. (2008). Cardiac changes in the intrauterine growth-restricted fetus. Semin. Perinatol. 32 (3), 190–193. 10.1053/j.semperi.2008.02.010 18482620

[B9] Barker D. J. (1990). The fetal and infant origins of adult disease. Bmj 301 (6761), 1111. 10.1136/bmj.301.6761.1111 2252919 PMC1664286

[B10] Barker D. J. (2006). Adult consequences of fetal growth restriction. Clin. Obstet. Gynecol. 49 (2), 270–283. 10.1097/00003081-200606000-00009 16721106

[B11] Berends L. M. Dearden L. Tung Y. C. L. Voshol P. Fernandez-Twinn D. S. Ozanne S. E. (2018). Programming of central and peripheral insulin resistance by low birthweight and postnatal catch-up growth in male mice. Diabetologia 61 (10), 2225–2234. 10.1007/s00125-018-4694-z 30043179 PMC6133152

[B12] Black M. J. Lim K. Zimanyi M. A. Sampson A. K. Bubb K. J. Flower R. L. (2015). Accelerated age-related decline in renal and vascular function in female rats following early-life growth restriction. Am. J. Physiol. Regul. Integr. Comp. Physiol. 309 (9), R1153–R1161. 10.1152/ajpregu.00403.2014 26377562

[B13] Black R. E. (2015). Nestle nutr inst workshop ser, 81, 1–7. 10.1159/000365790 26111558

[B14] Botting K. J. Loke X. Y. Zhang S. Andersen J. B. Nyengaard J. R. Morrison J. L. (2018). IUGR decreases cardiomyocyte endowment and alters cardiac metabolism in a sex- and cause-of-IUGR-specific manner. Am. J. Physiol. Regul. Integr. Comp. Physiol. 315 (1), R48–r67. 10.1152/ajpregu.00180.2017 29561647

[B15] Butruille L. Mayeur S. Duparc T. Knauf C. Moitrot E. Fajardy I. (2012). Prenatal fasudil exposure alleviates fetal growth but programs hyperphagia and overweight in the adult male rat. Eur. J. Pharmacol. 689 (1-3), 278–284. 10.1016/j.ejphar.2012.05.040 22683867

[B16] Canoy D. Pouta A. Ruokonen A. Hartikainen A. L. Saikku P. Järvelin M. R. (2009). Weight at birth and infancy in relation to adult leukocyte count: a population-based study of 5619 men and women followed from the fetal period to adulthood. J. Clin. Endocrinol. Metab. 94 (6), 1916–1922. 10.1210/jc.2008-2545 19276227

[B17] Carr D. J. Wallace J. M. Aitken R. P. Milne J. S. Martin J. F. Zachary I. C. (2016). Peri- and postnatal effects of prenatal adenoviral VEGF gene therapy in growth-restricted sheep. Biol. Reprod. 94 (6), 142. 10.1095/biolreprod.115.133744 27103444

[B18] Carr D. J. Wallace J. M. Aitken R. P. Milne J. S. Mehta V. Martin J. F. (2014). Uteroplacental adenovirus vascular endothelial growth factor gene therapy increases fetal growth velocity in growth-restricted sheep pregnancies. Hum. Gene Ther. 25 (4), 375–384. 10.1089/hum.2013.214 24593228 PMC3997090

[B19] Cheema K. K. Dent M. R. Saini H. K. Aroutiounova N. Tappia P. S. (2005). Prenatal exposure to maternal undernutrition induces adult cardiac dysfunction. Br. J. Nutr. 93 (4), 471–477. 10.1079/bjn20041392 15946408

[B20] Chen L. Yue J. Wu H. Yang J. Han X. Li J. (2015). Ouabain attenuates cardiac hypertrophy of male rat offspring exposed to intrauterine growth restriction following high-salt diet challenge. Reprod. Sci. 22 (12), 1587–1596. 10.1177/1933719115589412 26071389

[B21] Cheong J. N. Wlodek M. E. Moritz K. M. Cuffe J. S. (2016). Programming of maternal and offspring disease: impact of growth restriction, fetal sex and transmission across generations. J. Physiol. 594 (17), 4727–4740. 10.1113/jp271745 26970222 PMC5009791

[B22] Christie M. J. Romano T. Murphy R. M. Posterino G. S. (2018). The effect of intrauterine growth restriction on Ca(2+) -activated force and contractile protein expression in the mesenteric artery of adult (6-month-old) male and female Wistar-Kyoto rats. Physiol. Rep. 6 (24), e13954. 10.14814/phy2.13954 30592188 PMC6308111

[B23] Chu A. Casero D. Thamotharan S. Wadehra M. Cosi A. Devaskar S. U. (2019). The placental transcriptome in late gestational hypoxia resulting in murine intrauterine growth restriction parallels increased risk of adult cardiometabolic disease. Sci. Rep. 9 (1), 1243. 10.1038/s41598-018-37627-y 30718791 PMC6361888

[B24] Crispi F. Bijnens B. Figueras F. Bartrons J. Eixarch E. Le Noble F. (2010). Fetal growth restriction results in remodeled and less efficient hearts in children. Circulation 121 (22), 2427–2436. 10.1161/circulationaha.110.937995 20497977

[B25] Curhan G. C. Chertow G. M. Willett W. C. Spiegelman D. Colditz G. A. Manson J. E. (1996). Birth weight and adult hypertension and obesity in women. Circulation 94 (6), 1310–1315. 10.1161/01.cir.94.6.1310 8822985

[B26] Dimasi C. G. Darby J. R. T. (2022). Does the growth restricted fetal heart burn less fat? J. Physiol. 600 (7), 1585–1586. 10.1113/jp282900 35218562

[B27] Dimasi C. G. Darby J. R. T. Cho S. K. S. Saini B. S. Holman S. L. Meakin A. S. (2023). Reduced *in utero* substrate supply decreases mitochondrial abundance and alters the expression of metabolic signalling molecules in the fetal sheep heart. J. Physiol. 602 (eng), 5901–5922. 10.1113/jp285572 37996982

[B28] Dimasi C. G. Lazniewska J. Plush S. E. Saini B. S. Holman S. L. Cho S. K. S. (2021). Redox ratio in the left ventricle of the growth restricted fetus is positively correlated with cardiac output. J. Biophot. 14 (12), e202100157. 10.1002/jbio.202100157 34499415

[B29] Dodson R. B. Rozance P. J. Fleenor B. S. Petrash C. C. Shoemaker L. G. Hunter K. S. (2013). Increased arterial stiffness and extracellular matrix reorganization in intrauterine growth-restricted fetal sheep. Pediatr. Res. 73 (2), 147–154. 10.1038/pr.2012.156 23154756 PMC3742323

[B30] Drake R. R. Louey S. Thornburg K. L. (2022). Intrauterine growth restriction elevates circulating acylcarnitines and suppresses fatty acid metabolism genes in the fetal sheep heart. J. Physiol. 600 (3), 655–670. 10.1113/jp281415 34802149 PMC9075772

[B31] Drever N. Saade G. R. Bytautiene E. (2010). Fetal programming: early-life modulations that affect adult outcomes. Curr. Allergy Asthma Rep. 10 (6), 453–459. 10.1007/s11882-010-0136-9 20617403

[B32] Fu L. C. Lv Y. Zhong Y. He Q. Liu X. Du L. Z. (2017). Tyrosine phosphorylation of Kv1.5 is upregulated in intrauterine growth retardation rats with exaggerated pulmonary hypertension. Braz J. Med. Biol. Res. 50 (11), e6237. 10.1590/1414-431x20176237 28902925 PMC5597283

[B33] Golic M. Stojanovska V. Bendix I. Wehner A. Herse F. Haase N. (2018). Diabetes mellitus in pregnancy leads to growth restriction and epigenetic modification of the Srebf2 gene in rat fetuses. Hypertension 71 (5), 911–920. 10.1161/hypertensionaha.117.10782 29610268

[B34] Graupner O. Ried C. Wildner N. K. Ortiz J. U. Kuschel B. Haller B. (2022). Myocardial deformation analysis in late-onset small-for-gestational-age and growth-restricted fetuses using two-dimensional speckle tracking echocardiography: a prospective cohort study. J. Perinat. Med. 50 (3), 305–312. 10.1515/jpm-2021-0162 34529908

[B35] Guillot E. Lemay A. Allouche M. Vitorino Silva S. Coppola H. Sabatier F. (2023). Resveratrol reverses endothelial colony-forming cell dysfunction in adulthood in a rat model of intrauterine growth restriction. Int. J. Mol. Sci. 24 (11), 9747. 10.3390/ijms24119747 37298697 PMC10253508

[B36] Guitart-Mampel M. Juarez-Flores D. L. Youssef L. Moren C. Garcia-Otero L. Roca-Agujetas V. (2019). Mitochondrial implications in human pregnancies with intrauterine growth restriction and associated cardiac remodelling. J. Cell Mol. Med. 23 (6), 3962–3973. 10.1111/jcmm.14282 30941904 PMC6533501

[B37] Gutiérrez-Arzapalo P. Y. Rodríguez-Rodríguez P. Ramiro-Cortijo D. López de Pablo Á. L. López-Giménez M. R. Condezo-Hoyos L. (2018). Role of fetal nutrient restriction and postnatal catch-up growth on structural and mechanical alterations of rat aorta. J. Physiol. 596 (23), 5791–5806. 10.1113/jp275030 29277911 PMC6265550

[B38] Hansell J. A. Richter H. G. Camm E. J. Herrera E. A. Blanco C. E. Villamor E. (2022). Maternal melatonin: effective intervention against developmental programming of cardiovascular dysfunction in adult offspring of complicated pregnancy. J. Pineal Res. 72 (1), e12766. 10.1111/jpi.12766 34634151

[B39] He Q. Liu X. Zhong Y. Xu S. S. Zhang Z. M. Tang L. L. (2018). Arginine bioavailability and endothelin-1 system in the regulation of vascular function of umbilical vein endothelial cells from intrauterine growth restricted newborns. Nutr. Metab. Cardiovasc Dis. 28 (12), 1285–1295. 10.1016/j.numecd.2018.09.002 30392707

[B40] Iodice S. Hoxha M. Ferrari L. Carbone I. F. Anceschi C. Miragoli M. (2018). Particulate air pollution, blood mitochondrial DNA copy number, and telomere length in mothers in the first trimester of pregnancy: effects on fetal growth. Oxid. Med. Cell Longev. 2018, 5162905. 10.1155/2018/5162905 30524658 PMC6247572

[B41] Itani N. Skeffington K. L. Beck C. Niu Y. Giussani D. A. (2016). Melatonin rescues cardiovascular dysfunction during hypoxic development in the chick embryo. J. Pineal Res. 60 (1), 16–26. 10.1111/jpi.12283 26444711 PMC4832387

[B42] Jiang R. Teng Y. Huang Y. Gu J. Ma L. Li M. (2014). Preeclampsia serum-induced collagen I expression and intracellular calcium levels in arterial smooth muscle cells are mediated by the PLC-γ1 pathway. Exp. Mol. Med. 46 (9), e115. 10.1038/emm.2014.59 25257609 PMC4183944

[B43] Jonker S. S. Louey S. Roselli C. E. (2018). Cardiac myocyte proliferation and maturation near term is inhibited by early gestation maternal testosterone exposure. Am. J. Physiol. Heart Circ. Physiol. 315 (5), H1393–h1401. 10.1152/ajpheart.00314.2018 30095996 PMC6297822

[B44] Kesavan K. Devaskar S. U. (2019). Intrauterine growth restriction: postnatal monitoring and outcomes. Pediatr. Clin. North Am. 66 (2), 403–423. 10.1016/j.pcl.2018.12.009 30819345

[B45] Kimura C. Watanabe K. Iwasaki A. Mori T. Matsushita H. Shinohara K. (2013). The severity of hypoxic changes and oxidative DNA damage in the placenta of early-onset preeclamptic women and fetal growth restriction. J. Matern. Fetal Neonatal Med. 26 (5), 491–496. 10.3109/14767058.2012.733766 23035823

[B46] Krause B. J. Peñaloza E. Candia A. Cañas D. Hernández C. Arenas G. A. (2019). Adult vascular dysfunction in foetal growth-restricted Guinea-pigs is associated with a neonate-adult switching in Nos3 DNA methylation. Acta Physiol. (Oxf) 227 (3), e13328. 10.1111/apha.13328 31177629

[B47] Kumar P. Morton J. S. Shah A. Do V. Sergi C. Serrano-Lomelin J. (2020). Intrauterine exposure to chronic hypoxia in the rat leads to progressive diastolic function and increased aortic stiffness from early postnatal developmental stages. Physiol. Rep. 8 (1), e14327. 10.14814/phy2.14327 31960611 PMC6971413

[B48] Kuo A. H. Li C. Huber H. F. Schwab M. Nathanielsz P. W. Clarke G. D. (2017a). Maternal nutrient restriction during pregnancy and lactation leads to impaired right ventricular function in young adult baboons. J. Physiol. 595 (13), 4245–4260. 10.1113/jp273928 28439937 PMC5491873

[B49] Kuo A. H. Li C. Li J. Huber H. F. Nathanielsz P. W. Clarke G. D. (2017b). Cardiac remodelling in a baboon model of intrauterine growth restriction mimics accelerated ageing. J. Physiol. 595 (4), 1093–1110. 10.1113/jp272908 27988927 PMC5309359

[B50] Küster A. Croyal M. Moyon T. Darmaun D. Ouguerram K. Ferchaud-Roucher V. (2023). Characterization of lipoproteins and associated lipidome in very preterm infants: a pilot study. Pediatr. Res. 93 (4), 938–947. 10.1038/s41390-022-02159-9 35739258

[B51] Lane S. L. Houck J. A. Doyle A. S. Bales E. S. Lorca R. A. Julian C. G. (2020). AMP-activated protein kinase activator AICAR attenuates hypoxia-induced murine fetal growth restriction in part by improving uterine artery blood flow. J. Physiol. 598 (18), 4093–4105. 10.1113/jp279341 32592403 PMC7749723

[B52] Law C. M. Shiell A. W. (1996). Is blood pressure inversely related to birth weight? The strength of evidence from a systematic review of the literature. J. Hypertens. 14 (8), 935–942. 10.1097/00004872-199608000-00002 8884547

[B53] Leon D. A. Johansson M. Rasmussen F. (2000). Gestational age and growth rate of fetal mass are inversely associated with systolic blood pressure in young adults: an epidemiologic study of 165,136 Swedish men aged 18 years. Am. J. Epidemiol. 152 (7), 597–604. 10.1093/aje/152.7.597 11032153

[B54] Leon D. A. Lithell H. O. Vâgerö D. Koupilová I. Mohsen R. Berglund L. (1998). Reduced fetal growth rate and increased risk of death from ischaemic heart disease: cohort study of 15 000 Swedish men and women born 1915-29. Bmj 317 (7153), 241–245.eng. 10.1136/bmj.317.7153.241 9677213 PMC28614

[B55] Li M. Zhang Z. Joynauth J. Zhan X. Du L. (2022b). Intrauterine growth restriction neonates present with increased angiogenesis through the Notch1 signaling pathway. Microvasc. Res. 140, 104308. 10.1016/j.mvr.2021.104308 34995552

[B56] Li P. He L. Lan Y. Fang J. Fan Z. Li Y. (2022a). Intrauterine growth restriction induces adulthood chronic metabolic disorder in cardiac and skeletal muscles. Front. Nutr. 9, 929943. 10.3389/fnut.2022.929943 35938117 PMC9354130

[B57] Liu X. Qi Y. Tian B. Chen D. Gao H. Xi C. (2014). Maternal protein restriction induces alterations in hepatic tumor necrosis factor-α/CYP7A1 signaling and disorders regulation of cholesterol metabolism in the adult rat offspring. J. Clin. Biochem. Nutr. 55 (1), 40–47. 10.3164/jcbn.13-100 25120278 PMC4078062

[B58] Liu Y. Un E. M. Bai Y. Chan M. K. Zeng L. X. Lei S. L. (2024). Safety and efficacy of phosphodiesterase-5 (PDE-5) inhibitors in fetal growth restriction: a systematic literature review and meta-analysis. J. Pharm. Pharm. Sci. 27, 13206. 10.3389/jpps.2024.13206 39211421 PMC11357966

[B59] Lopez-Tello J. Arias-Alvarez M. Gonzalez-Bulnes A. Sferuzzi-Perri A. N. (2019). Models of Intrauterine growth restriction and fetal programming in rabbits. Mol. Reprod. Dev. 86 (12), 1781–1809. 10.1002/mrd.23271 31538701

[B60] Lv Y. Fu L. Zhang Z. Gu W. Luo X. Zhong Y. (2019). Increased expression of MicroRNA-206 inhibits potassium voltage-gated channel subfamily A member 5 in pulmonary arterial smooth muscle cells and is related to exaggerated pulmonary artery hypertension following intrauterine growth retardation in rats. J. Am. Heart Assoc. 8 (2), e010456. 10.1161/jaha.118.010456 30636484 PMC6497345

[B61] Mankouski A. Miller T. A. Dodson R. B. Yu B. Yang Y. Liu J. (2022). Large artery stiffening and mortality in a rat model of early vascular remodeling induced by intrauterine growth restriction and a high-fat diet. Physiol. Rep. 10 (23), e15518. 10.14814/phy2.15518 36461654 PMC9718947

[B62] Marciniak A. Patro-Małysza J. Kimber-Trojnar Ż. Marciniak B. Oleszczuk J. Leszczyńska-Gorzelak B. (2017). Fetal programming of the metabolic syndrome. Taiwan J. Obstet. Gynecol. 56 (2), 133–138. 10.1016/j.tjog.2017.01.001 28420495

[B63] Martyn C. N. Barker D. J. Jespersen S. Greenwald S. Osmond C. Berry C. (1995). Growth *in utero*, adult blood pressure, and arterial compliance. Br. Heart J. 73 (2), 116–121. 10.1136/hrt.73.2.116 7696018 PMC483775

[B64] Masi S. Jones A. Charakida M. O'Neill F. D'Aiuto F. Virdis A. (2011). Blood pressure and vascular alterations with growth in childhood. Curr. Pharm. Des. 17 (28), 3045–3061. 10.2174/138161211798157757 21861832

[B65] McMillen I. C. Robinson J. S. (2005). Developmental origins of the metabolic syndrome: prediction, plasticity, and programming. Physiol. Rev. 85(2):571–633. 10.1152/physrev.00053.2003 15788706

[B66] Menendez-Castro C. Toka O. Fahlbusch F. Cordasic N. Wachtveitl R. Hilgers K. F. (2014). Impaired myocardial performance in a normotensive rat model of intrauterine growth restriction. Pediatr. Res. 75 (6), 697–706. 10.1038/pr.2014.27 24603294

[B67] Mills V. Plows J. F. Zhao H. Oyston C. Vickers M. H. Baker P. N. (2018). Effect of sildenafil citrate treatment in the eNOS knockout mouse model of fetal growth restriction on long-term cardiometabolic outcomes in male offspring. Pharmacol. Res. 137, 122–134. 10.1016/j.phrs.2018.09.023 30292428

[B68] Nevin C. L. Formosa E. Maki Y. Matushewski B. Regnault T. R. H. Richardson B. S. (2018). Maternal nutrient restriction in Guinea pigs as an animal model for studying growth-restricted offspring with postnatal catch-up growth. Am. J. Physiol. Regul. Integr. Comp. Physiol. 314 (5), R647–r654. 10.1152/ajpregu.00317.2017 29351419

[B69] Nilsson P. M. Ostergren P. O. Nyberg P. Söderström M. Allebeck P. (1997). Low birth weight is associated with elevated systolic blood pressure in adolescence: a prospective study of a birth cohort of 149378 Swedish boys. J. Hypertens. 15 (12 Pt 2), 1627–1631. 10.1097/00004872-199715120-00064 9488214

[B70] Ojeda N. B. Royals T. P. Alexander B. T. (2013). Sex differences in the enhanced responsiveness to acute angiotensin II in growth-restricted rats: role of fasudil, a Rho kinase inhibitor. Am. J. Physiol. Ren. Physiol. 304 (7), F900–F907. 10.1152/ajprenal.00687.2012 PMC3625844 23344570

[B71] Oliveira B. Flôr DELF Rocha G. Rodrigues M. Ladeiras R. Guimarães H. (2021). The impact of intrauterine growth restriction on respiratory outcomes. Minerva Pediatr. (Torino) 73 (5), 426–434. 10.23736/s2724-5276.17.04965-9 28565900

[B72] Oliveira V. de Souza L. V. Fernandes T. Junior S. D. S. de Carvalho M. H. C. Akamine E. H. (2017b). Intrauterine growth restriction-induced deleterious adaptations in endothelial progenitor cells: possible mechanism to impair endothelial function. J. Dev. Orig. Health Dis. 8 (6), 665–673. 10.1017/s2040174417000484 28689502

[B73] Oliveira V. Silva Junior S. D. de Carvalho M. H. Akamine E. H. Michelini L. C. Franco M. C. (2017a). Intrauterine growth restriction increases circulating mitochondrial DNA and Toll-like receptor 9 expression in adult offspring: could aerobic training counteract these adaptations? J. Dev. Orig. Health Dis. 8 (2), 236–243. 10.1017/s2040174416000714 28004624

[B74] Paules C. Dantas A. P. Miranda J. Crovetto F. Eixarch E. Rodriguez-Sureda V. (2019). Premature placental aging in term small-for-gestational-age and growth-restricted fetuses. Ultrasound Obstet. Gynecol. 53 (5), 615–622. 10.1002/uog.20103 30125412

[B75] Paz A. A. Arenas G. A. Castillo-Galán S. Peñaloza E. Cáceres-Rojas G. Suazo J. (2019). Premature vascular aging in Guinea pigs affected by fetal growth restriction. Int. J. Mol. Sci. 20 (14), eng. 10.3390/ijms20143474 PMC6678381 31311132

[B76] Pecks U. Bornemann V. Klein A. Segger L. Maass N. Alkatout I. (2019). Estimating fetal cholesterol synthesis rates by cord blood analysis in intrauterine growth restriction and normally grown fetuses. Lipids Health Dis. 18 (1), 185. 10.1186/s12944-019-1117-1 31653257 PMC6815065

[B77] Pereira S. P. Diniz M. S. Tavares L. C. Cunha-Oliveira T. Li C. Cox L. A. (2023). Characterizing early cardiac metabolic programming via 30% maternal nutrient reduction during fetal development in a non-human primate model. Int. J. Mol. Sci. 24 (20), 15192. 10.3390/ijms242015192 37894873 PMC10607248

[B78] Pereira S. P. Tavares L. C. Duarte A. I. Baldeiras I. Cunha-Oliveira T. Martins J. D. (2021). Sex-dependent vulnerability of fetal nonhuman primate cardiac mitochondria to moderate maternal nutrient reduction. Clin. Sci. (Lond). 135 (9), 1103–1126. 10.1042/cs20201339 33899910 PMC8456369

[B79] Peyter A. C. Armengaud J. B. Guillot E. Yzydorczyk C. (2021). Endothelial progenitor cells dysfunctions and cardiometabolic disorders: from mechanisms to therapeutic approaches. Int. J. Mol. Sci. 22 (13), 6667. 10.3390/ijms22136667 34206404 PMC8267891

[B80] Peyter A. C. Delhaes F. Baud D. Vial Y. Diaceri G. Menétrey S. (2014). Intrauterine growth restriction is associated with structural alterations in human umbilical cord and decreased nitric oxide-induced relaxation of umbilical vein. Placenta 35 (11), 891–899. 10.1016/j.placenta.2014.08.090 25249153

[B81] Pike K. C. Hanson M. A. Godfrey K. M. (2008). Developmental mismatch: consequences for later cardiorespiratory health. Bjog 115 (2), 149–157. 10.1111/j.1471-0528.2007.01603.x 18081597

[B82] Rakhanova Y. Almawi W. Y. Aimagambetova G. Riethmacher D. (2023). The effects of sildenafil citrate on intrauterine growth restriction: a systematic review and meta-analysis. BMC Pregnancy Childbirth 23 (1), 409. 10.1186/s12884-023-05747-7 37268873 PMC10236759

[B83] Rasmussen J. M. Thompson P. M. Entringer S. Buss C. Wadhwa P. D. (2022). Fetal programming of human energy homeostasis brain networks: issues and considerations. Obes. Rev. 23 (3), e13392. 10.1111/obr.13392 34845821 PMC10308600

[B84] Reyes L. M. Kirschenman R. Quon A. Morton J. S. Shah A. Davidge S. T. (2015). Aerobic exercise training reduces cardiac function in adult male offspring exposed to prenatal hypoxia. Am. J. Physiol. Regul. Integr. Comp. Physiol. 309 (5), R489–R498. 10.1152/ajpregu.00201.2015 26157059

[B85] Reyes L. M. Shah A. Quon A. Morton J. S. Davidge S. T. (2018). The role of the tumor necrosis factor (TNF)-related weak inducer of apoptosis (TWEAK) in offspring exposed to prenatal hypoxia. J. Dev. Orig. Health Dis. 9 (6), 661–669. 10.1017/s2040174417001003 29249219

[B86] Reynolds L. P. Borowicz P. P. Caton J. S. Crouse M. S. Dahlen C. R. Ward A. K. (2019). Developmental programming of fetal growth and development. Vet. Clin. North Am. Food Anim. Pract. 35 (2), 229–247. 10.1016/j.cvfa.2019.02.006 31103178

[B87] Rock C. R. White T. A. Piscopo B. R. Sutherland A. E. Miller S. L. Camm E. J. (2021). Cardiovascular and cerebrovascular implications of growth restriction: mechanisms and potential treatments. Int. J. Mol. Sci. 22 (14), 7555. 10.3390/ijms22147555 34299174 PMC8303639

[B88] Roifman M. Choufani S. Turinsky A. L. Drewlo S. Keating S. Brudno M. (2016). Genome-wide placental DNA methylation analysis of severely growth-discordant monochorionic twins reveals novel epigenetic targets for intrauterine growth restriction. Clin. Epigenetics 8, 70. 10.1186/s13148-016-0238-x 27330572 PMC4915063

[B89] Rossi P. Tauzin L. Marchand E. Boussuges A. Gaudart J. Frances Y. (2011). Respective roles of preterm birth and fetal growth restriction in blood pressure and arterial stiffness in adolescence. J. Adolesc. Health 48 (5), 520–522. 10.1016/j.jadohealth.2010.08.004 21501813

[B90] Scherrer U. Rimoldi S. F. Sartori C. Messerli F. H. Rexhaj E. (2015). Fetal programming and epigenetic mechanisms in arterial hypertension. Curr. Opin. Cardiol. 30 (4), 393–397. 10.1097/hco.0000000000000192 26049388

[B91] Sehgal A. Alexander B. T. Morrison J. L. South A. M. (2020). Fetal growth restriction and hypertension in the offspring: mechanistic links and therapeutic directions. J. Pediatr. 224, 115–123.e2. 10.1016/j.jpeds.2020.05.028 32450071 PMC8086836

[B92] Sehgal A. Allison B. J. Crispi F. Menahem S. (2023). Influence of accelerated arterial aging in growth-restricted cohorts on adult-onset cardiovascular diseases. Am. J. Physiol. Heart Circ. Physiol. 325 (1), H89–h105. 10.1152/ajpheart.00134.2023 37204872

[B93] Selivanova E. K. Shvetsova A. A. Shilova L. D. Tarasova O. S. Gaynullina D. K. (2021). Intrauterine growth restriction weakens anticontractile influence of NO in coronary arteries of adult rats.Sci. Rep. 11(1):14475. 10.1038/s41598-021-93491-3 34262070 PMC8280217

[B94] Severi F. M. Rizzo G. Bocchi C. D'Antona D. Verzuri M. S. Arduini D. (2000). Intrauterine growth retardation and fetal cardiac function. Fetal Diagn Ther. 15 (1), 8–19. 10.1159/000020969 10705209

[B95] Shah A. Quon A. Morton J. S. Davidge S. T. (2017). Postnatal resveratrol supplementation improves cardiovascular function in male and female intrauterine growth restricted offspring. Physiol. Rep. 5 (2), e13109. 10.14814/phy2.13109 28108646 PMC5269411

[B96] Shehata N. A. A. Ali H. A. A. Fahim A. S. Katta M. A. Hussein G. K. (2020). Addition of sildenafil citrate for treatment of severe intrauterine growth restriction: a double blind randomized placebo controlled trial. J. Matern. Fetal Neonatal Med. 33 (10), 1631–1637. 10.1080/14767058.2018.1523892 30345864

[B97] Simoncini S. Coppola H. Rocca A. Bachmann I. Guillot E. Zippo L. (2021). Endothelial colony-forming cells dysfunctions are associated with arterial hypertension in a rat model of intrauterine growth restriction. Int. J. Mol. Sci. 22 (18), 10159. 10.3390/ijms221810159 34576323 PMC8465555

[B98] Spiroski A. M. Niu Y. Nicholas L. M. Austin-Williams S. Camm E. J. Sutherland M. R. (2021). Mitochondria antioxidant protection against cardiovascular dysfunction programmed by early-onset gestational hypoxia. Faseb J. 35 (5), e21446. 10.1096/fj.202002705R 33788974 PMC7612077

[B99] Sutovska H. Babarikova K. Zeman M. Molcan L. (2022). Prenatal hypoxia affects foetal cardiovascular regulatory mechanisms in a sex- and circadian-dependent manner: a review. Int. J. Mol. Sci. 23 (5), 2885. 10.3390/ijms23052885 35270026 PMC8910900

[B100] Tang L. L. Zhang L. Y. Lao L. J. Hu Q. Y. Gu W. Z. Fu L. C. (2015). Epigenetics of Notch1 regulation in pulmonary microvascular rarefaction following extrauterine growth restriction. Respir. Res. 16 (1), 66. 10.1186/s12931-015-0226-2 26040933 PMC4486133

[B101] Tare M. Miller S. L. Wallace E. M. Sutherland A. E. Yawno T. Coleman H. A. (2012). Glucocorticoid treatment does not alter early cardiac adaptations to growth restriction in preterm sheep fetuses. Bjog 119 (8), 906–914. 10.1111/j.1471-0528.2012.03309.x 22703419

[B102] Tarry-Adkins J. L. Fernandez-Twinn D. S. Hargreaves I. P. Neergheen V. Aiken C. E. Martin-Gronert M. S. (2016). Coenzyme Q10 prevents hepatic fibrosis, inflammation, and oxidative stress in a male rat model of poor maternal nutrition and accelerated postnatal growth. Am. J. Clin. Nutr. 103 (2), 579–588. 10.3945/ajcn.115.119834 26718412 PMC4733260

[B103] Terstappen F. Calis J. J. A. Paauw N. D. Joles J. A. van Rijn B. B. Mokry M. (2020). Developmental programming in human umbilical cord vein endothelial cells following fetal growth restriction. Clin. Epigenetics 12 (1), 185. 10.1186/s13148-020-00980-9 33256815 PMC7708922

[B104] Terstappen F. Spradley F. T. Bakrania B. A. Clarke S. M. Joles J. A. Paauw N. D. (2019). Prenatal sildenafil therapy improves cardiovascular function in fetal growth restricted offspring of dahl salt-sensitive rats. Hypertension 73 (5), 1120–1127. 10.1161/hypertensionaha.118.12454 30827146 PMC6458081

[B105] Tintu A. Rouwet E. Verlohren S. Brinkmann J. Ahmad S. Crispi F. (2009). Hypoxia induces dilated cardiomyopathy in the chick embryo: mechanism, intervention, and long-term consequences. PLoS One 4 (4), e5155. 10.1371/journal.pone.0005155 19357774 PMC2663815

[B106] Ushida T. Cotechini T. Protopapas N. Atallah A. Collyer C. Toews A. J. (2022b). Aberrant inflammation in rat pregnancy leads to cardiometabolic alterations in the offspring and intrauterine growth restriction in the F2 generation. J. Dev. Orig. Health Dis. 13 (6), 706–718. 10.1017/s2040174422000265 35593438

[B107] Ushida T. Kotani T. Nakatochi M. Kobayashi Y. Nakamura N. Imai K. (2022a). Intrauterine exposure to hypertensive disorders of pregnancy and postnatal growth in extremely and very preterm infants. Pregnancy Hypertens. 28, 174–179. 10.1016/j.preghy.2022.05.007 35569242

[B108] Verschuren M. T. Morton J. S. Abdalvand A. Mansour Y. Rueda-Clausen C. F. Compston C. A. (2012). The effect of hypoxia-induced intrauterine growth restriction on renal artery function. J. Dev. Orig. Health Dis. 3 (5), 333–341. 10.1017/s2040174412000268 25102262

[B109] Visentin S. Grisan E. Zanardo V. Bertin M. Veronese E. Cavallin F. (2013). Developmental programming of cardiovascular risk in intrauterine growth-restricted twin fetuses according to aortic intima thickness. J. Ultrasound Med. 32 (2), 279–284. 10.7863/jum.2013.32.2.279 23341384

[B110] Visentin S. Grumolato F. Nardelli G. B. Di Camillo B. Grisan E. Cosmi E. (2014b). Early origins of adult disease: low birth weight and vascular remodeling. Atherosclerosis 237 (2), 391–399. 10.1016/j.atherosclerosis.2014.09.027 25463063

[B111] Visentin S. Lapolla A. Londero A. P. Cosma C. Dalfrà M. Camerin M. (2014a). Adiponectin levels are reduced while markers of systemic inflammation and aortic remodelling are increased in intrauterine growth restricted mother-child couple. Biomed. Res. Int. 2014, 401595. 10.1155/2014/401595 25045669 PMC4090565

[B112] Voggel J. Lubomirov L. Lechner F. Fink G. Nüsken E. Wohlfarth M. (2021). Vascular tone regulation in renal interlobar arteries of male rats is dysfunctional after intrauterine growth restriction. Am. J. Physiol. Ren. Physiol. 321 (1), F93–f105. 10.1152/ajprenal.00653.2020 34056927

[B113] von Ehr J. von Versen-Höynck F. (2016). Implications of maternal conditions and pregnancy course on offspring's medical problems in adult life. Arch. Gynecol. Obstet. 294 (4), 673–679. 10.1007/s00404-016-4178-7 27522600

[B114] Vranas S. Heinemann G. K. Liu H. De Blasio M. J. Owens J. A. Gatford K. L. (2017). Small size at birth predicts decreased cardiomyocyte number in the adult ovine heart. J. Dev. Orig. Health Dis. 8 (5), 618–625. 10.1017/s2040174417000381 28975880

[B115] Wang Q. Zhang Y. Zhou R. (2023). Expectant management for umbilical artery thrombosis in monochorionic diamniotic twin pregnancies: a case report. BMC Pregnancy Childbirth 23 (1), 515. 10.1186/s12884-023-05834-9 37452280 PMC10347762

[B116] Weatherall E. L. Avilkina V. Cortes-Araya Y. Dan-Jumbo S. Stenhouse C. Donadeu F. X. (2020). Differentiation potential of mesenchymal stem/stromal cells is altered by intrauterine growth restriction. Front. Vet. Sci. 7, 558905. 10.3389/fvets.2020.558905 33251256 PMC7676910

[B117] Wohlmuth C. Agarwal A. Stevens B. Johnson A. Moise K. J. Jr. Papanna R. (2020). Fetal ventricular strain in uncomplicated and selective growth-restricted monochorionic diamniotic twin pregnancies and cardiovascular response in pre-twin-twin transfusion syndrome. Ultrasound Obstet. Gynecol. 56 (5), 694–704. 10.1002/uog.21911 31682302 PMC7702120

[B118] Xu X. F. Lv Y. Gu W. Z. Tang L. L. Wei J. K. Zhang L. Y. (2013). Epigenetics of hypoxic pulmonary arterial hypertension following intrauterine growth retardation rat: epigenetics in PAH following IUGR. Respir. Res. 14 (1), 20. 10.1186/1465-9921-14-20 23406533 PMC3577465

[B119] Yamauchi T. Mogi M. Kan-No H. Shan B. S. Higaki A. Min L. J. (2018). Roles of angiotensin II type 2 receptor in mice with fetal growth restriction. Hypertens. Res. 41 (3), 157–164. 10.1038/s41440-017-0004-2 29335616

[B120] Yan L. Wang Y. Zhang Z. Xu S. Ullah R. Luo X. (2019). Postnatal delayed growth impacts cognition but rescues programmed impaired pulmonary vascular development in an IUGR rat model. Nutr. Metab. Cardiovasc Dis. 29 (12), 1418–1428. 10.1016/j.numecd.2019.08.016 31653519

[B121] Yates D. T. Camacho L. E. Kelly A. C. Steyn L. V. Davis M. A. Antolic A. T. (2019). Postnatal β2 adrenergic treatment improves insulin sensitivity in lambs with IUGR but not persistent defects in pancreatic islets or skeletal muscle. J. Physiol. 597 (24), 5835–5858. 10.1113/jp278726 31665811 PMC6911010

[B122] Yuliana M. E. Chou H. C. Su E. C. Chuang H. C. Huang L. T. Chen C. M. (2024). Uteroplacental insufficiency decreases leptin expression and impairs lung development in growth-restricted newborn rats. Pediatr Res. 95 (6), 1503–1509. 10.1038/s41390-023-02946-y 38049649

[B123] Zhou J. Zhu C. Luo H. Shen L. Gong J. Wu Y. (2019). Two intrauterine programming mechanisms of adult hypercholesterolemia induced by prenatal nicotine exposure in male offspring rats. Faseb J. 33 (1), 1110–1123. 10.1096/fj.201800172R 30113880

[B124] Zouridis A. Manousopoulou A. Potiris A. Sarli P. M. Aravantinos L. Pervanidou P. (2021). Impact of maternal food restriction on heart proteome in appropriately grown and growth-restricted wistar-rat offspring. Nutrients 13 (2), 466. 10.3390/nu13020466 33573223 PMC7912475

